# Editorial: Electrochemiluminescence: From Fundamentals to Applications

**DOI:** 10.3389/fchem.2021.706465

**Published:** 2021-05-31

**Authors:** Fabio Rizzo, Federico Polo, Neso Sojic, Guobao Xu

**Affiliations:** ^1^ Institute of Chemical Sciences and Technologies “G. Natta” (SCITEC), National Research Council (CNR), Milan, Italy; ^2^ Center for Soft Nanoscience (SON), Westfälische Wilhelms-Universität Münster, Münster, Germany; ^3^ Ca’ Foscari University of Venice, Venice, Italy; ^4^ University of Bordeaux, Bordeaux INP, CNRS, UMR 5255, Site ENSCBP, Pessac, France; ^5^ State Key Laboratory of Electroanalytical Chemistry, Changchun Institute of Applied Chemistry, Chinese Academy of Sciences, Changchun, China; ^6^ School of Applied Chemistry and Engineering, University of Science and Technology of China, Hefei, China

**Keywords:** electrochemiluminescence, transition metal complex, point-of-care system, halide perovskite, biosensing, nanomaterials, electrode array, imaging

Electrochemiluminescence (ECL) is a fascinating phenomenon arising from a highly energetic electron transfer reaction between electrogenerated radicals that generate an excited state species causing the emission of a photon. ECL was employed as powerful detection tool in the field of bioassays, which has gained increasing interest from both academic and industrial sectors. Indeed, it offers several advantages such as high sensitivity, very low background signal, and direct proportionality of the ECL intensity to the concentration of the luminophore species involved. Last but not least, along with the above mentioned traits, the relatively low-cost of the instrumentation required has helped to spread its popularity.

In recent years, the scientific community has developed a growing interest in ECL, as witnessed by the appearance of numerous innovative luminophores and an increasing number of analytical, imaging, and light-emitting applications. The topic of this Special Issue aims at including a collection of original research and review articles describing the application of novel dyes and the role of the electrode materials on the generation of the electrochemical light-emitting phenomenon.

The use of water-soluble dyes emitting at wavelengths other than that of ruthenium complexes is crucial for the development of the multicoloured ECL-sensors. In this respect, Newman et al. presented four different iridium complexes bearing tetraethylene-glycol (TEG) groups to enhance the solubility in aqueous solution. Besides the tuning of the emission between blue-greenish and orange, the TEG groups offer the possibility to act as binding site for bioconjugation, opening novel scenario for future development of ECL labels.

Nanomaterials are also attracting growing interest as ECL emitters. Zhang and Liu reviewed the recent development on the field of ECL nanoprobes by describing the structure-function relationship in different kind of inorganic nanomaterials, such as quantum dots, Au nanoclusters and 2D materials. Moreover, they emphasized the impact of the nanoprobes on the development of ECL imaging and multidimensional analysis. By following the guideline of this mini-review, readers can find some useful strategies for the design of future ECL nanoprobes. Among the inorganic nanomaterials that have been used as ECL emitters, halide perovskites represent an emerging and promising subject. In their mini-review, Cao and Zhu introduced the reader into the field of these inorganic nanomaterials from the basic characteristics to the ECL-mechanism, together with a brief description of synthetic approaches. Meanwhile, the authors discussed the strategy to enhance their stability as well as the role of the interfacial modification and the first studies on ECL sensing. The perovskites investigated for ECL purposes after the first report appeared in 2016 are here summarized, demonstrating the growing interest on these materials.

Electrodes play a pivotal role on the detection of ECL signal and on its applications in biosensing. To optimize the measurement of the ECL emission several features must be considered, such as the employed material, the active area and the shape. Inside this Special Issue three original research articles are focused on this important subject. Ding et al. reported on the fabrication of ultra-high-density gold microwell electrodes and their use to enhance the intensity of ECL signal. The experimental data are supported by theoretical simulation, resulting in an important contribution for the development of ECL imaging systems. Forster et al. presented the electrochemical and ECL studies of three-dimensional (3D) printed electrodes. The working electrode consisting of titanium microcylinder array is obtained by means of the Selective Laser Melting (SLM) technique, resulting in a cost-effective approach to produce precise and controlled structure in the micro and macro scale. Furthermore, the 3D-printed electrodes offer the possibility to electrogenerate light in small volumes instead of surfaces, resulting in an enhancement of the signal intensity in selected regions. Besides, the authors showed the possibility to electrodeposit a thin layer of gold onto the 3D printed electrode, which increases the rate of the heterogenous electron transfer process and, as a consequence, the ECL intensity. This study opens novel scenarios on the development of 3D printed materials with unusual properties. Another very interesting original contribution is made by Kerr et al., who analyzed a series of commercially available screen-printed electrodes (SPEs) as suitable systems for electrochemical and ECL detection. This study is expected to have a great impact on the experimental setup used for ECL analyses. In fact, they compared 13 commercially available SPEs made on traditional materials such as carbon, gold and platinum, and modified electrodes incorporating carbon nanofibers, carbon nanotubes, gold nanoparticles, graphene, ordered mesoporous carbon and combinations of these materials. Moreover, the authors showed that the lack of mechanical polishing limits the performance of SPEs to a single experiment, thus hampering the use of each SPE for more tests. The readers will surely benefit of these findings to choose the optimal SPEs for their application from the plethora of commercially available varieties of SPEs.

As Guest Editors of this topic issue, we would like to thank all the authors for their contributions and all the referees for their invaluable work, thoughtful suggestions and insights. We hope that this Special Issue will inspire the researchers worldwide and that future endeavors will contribute to the development of more efficient ECL systems and a wider variety of sensing applications.

